# PARP1-DNA co-condensation: the driver of broken DNA repair

**DOI:** 10.1038/s41392-024-01832-1

**Published:** 2024-05-17

**Authors:** Xiang Wei, Fangfang Zhou, Long Zhang

**Affiliations:** 1 Life Sciences Institute, The Second Affiliated Hospital of the Zhejiang University School of Medicine, The MOE Key Laboratory of Biosystems Homeostasis & Protection and Zhejiang Provincial Key Laboratory for Cancer Molecular Cell Biology, Zhejiang University, Hangzhou, China; 2 The First Affiliated Hospital, the Institutes of Biology and Medical Sciences, Suzhou Medical College, Soochow University, Suzhou, Jiangsu, 215123 China

**Keywords:** Senescence, Ageing

## Abstract

**Figure:**
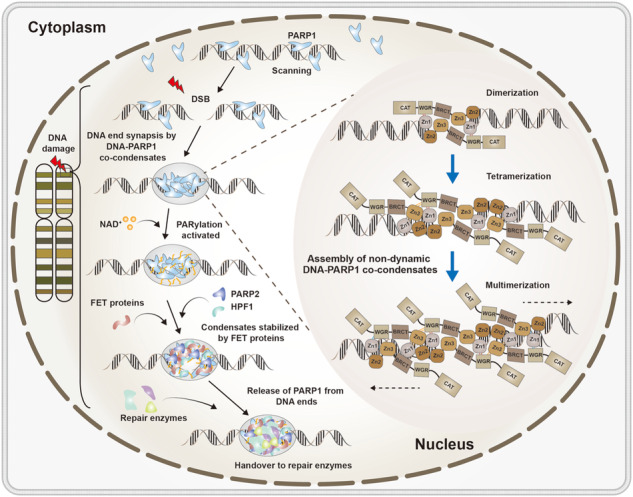
DNA double-strand break (DSB) sites that prevent the disjunction of broken DNA ends are formed through poly (ADP-ribose) (PAR) polymerase 1 (PARP1)-DNA co-condensation. The co-condensates apply mechanical forces to hold the DNA ends together and generate enzymatic activity for the synthesis of PAR. PARylation can promote the release of PARP1 from DNA ends and recruit various proteins, such as Fused in sarcoma (FUS) proteins, thereby stabilizing broken DNA ends and preventing their separation.

DNA double-strand break (DSB) sites that prevent the disjunction of broken DNA ends are formed through poly (ADP-ribose) (PAR) polymerase 1 (PARP1)-DNA co-condensation. The co-condensates apply mechanical forces to hold the DNA ends together and generate enzymatic activity for the synthesis of PAR. PARylation can promote the release of PARP1 from DNA ends and recruit various proteins, such as Fused in sarcoma (FUS) proteins, thereby stabilizing broken DNA ends and preventing their separation.

A recent study published by Simon Alberti^1^ in *Cell* has revealed that DNA double-strand break (DSB) sites that prevent the disjunction of broken DNA ends are formed through poly (ADP-ribose) (PAR) polymerase 1 (PARP1)-DNA co-condensation.^1^ (Fig. 1). Here, a comprehensive and novel model was provided for the ordered assembly of PARP1-DNA condensates, which can rationally make clear broken DNA end synapses and how repair effectors are recruited to repair DSB.

**Fig. 1:**
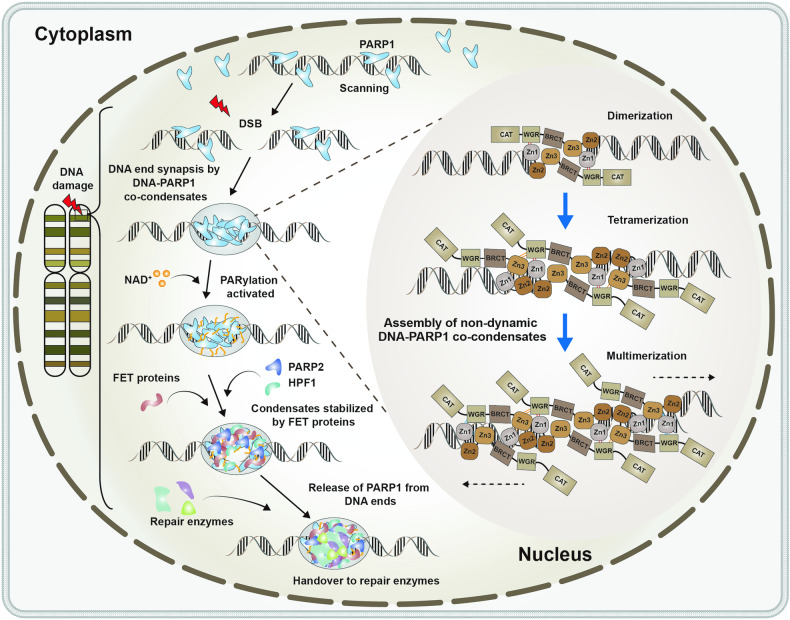
The mechanistic mode of PARP1-DNA co-condensation as the driver of broken DNA repair. The mechanistic model for the hierarchical assembly of DNA double-strand break (DSB) condensates to illustrating DNA end synapsis and the recruitment of effector proteins for DNA damage repair. Firstly, poly (ADP-ribose) (PAR) polymerase 1 (PARP1) dimerization and multimerization along DNA mediates co-condensate assembly. Then, rigid PARP1-DNA co-condensates generate forces and mediate synapsis of broken DNA ends. The condensates become enzymatically active and PARP1 PARylation destabilizes condensates. Finally, FET proteins stabilize condensates against dissolution, allowing for the entry of repair factors. (Fig. 1 is adapted from graphic abstract^1^)

Several types of damage in cells, including DNA-RNA hybridization, nucleotide base destruction and DNA strand breaks can occur due to either dangerous environmental exposure or normal cellular processes. Double-stranded (ds) DNA breaks are extremely devastating, causing the destruction of chromosomes and even cell death. There are two main approaches to repairing DSB and maintaining genomic stability: homologous recombination and non-homologous DNA end-joining.^2,3^ PARP1 makes a vast difference in detecting DNA damage and initiating repair pathways. PARP1 is highly concentrated at DSB sites, along with other DNA damage components, and participates in the assembly of DSB sites during DNA damage.^3^ However, a comprehensive understanding of how DSB sites are assembled, how broken DNA prevents segregation and eventually being repaired is currently lacking at the molecular level.

PARP1 is localized to the nucleus and is a highly abundant nuclear protein.^4^ To determine how PARP1 and DNA form condensates, the authors first purified full-length proteins in vitro to reconstruct DNA repair sites. Using biophysical bulk, single-molecule techniques, and quantitative biochemistry, it was demonstrated that PARP1 co-condenses with broken DNA, mediating DNA synapses at the ends of DNA lesions. The authors observed, in vitro, that the probability of co-condensation increased with the length of DNA by binding multiple PARP1 molecules. In contrast, homologous PARP2, single-stranded (ss) DNA, ss-RNA, and ds-RNA didn’t play roles in condensate formation. Blocking both the 5’ and 3’ DNA ends prevents condensate aggregation, blocking either of the two ends of one DNA strand reduces partial condensate aggregation, and blocking the one end of both DNA strands also impairs the aggregation of the condensate, elucidating that PARP1 interacts with both DNA strands, and PARP1 interacts with the 5 ‘end and 3’ end respectively to stabilize the condensate.

Based on extensive experimental results, the authors proposed a model in which PARP1 and damaged DNA ends co-condense to reconcile the junctions between the broken DNA ends. Mechanistically, the initial step is driven by the binding of zinc finger 1 (ZnF1) and ZnF2 of the PARP1 protein to DNA, whereas intermolecular interactions provided by the BRCA1 C-terminus (BRCT) and tryptophan-glycine-arginine-rich domains are required to keep the PARP1 dimer stable. Then, through intermolecular protein-protein interactions supplied by PARP1 molecules between the interfaces of the ZnF1, ZnF3, and BRCT domains, the tetramer and hexamer are further assembled to form PARP1-DNA co-condensates, which are established during DNA binding. These interactions within molecules provide balanced bidirectional cohesion to prevent DNA ends separation. The formation of PARP1-DNA co-condensates and PARP1 activation occur simultaneously. PARP1 PARylation occurs primarily in the C-terminal domain and destabilizes condensates. Crucially, PARylation dissociates PARP1 to expose DNA ends. Next, the PAR polymers begin to recruit other effectors, such as FET (FUS, EWSR1 (Ewing sarcoma breakpoint region 1/EWS RNA binding protein 1), and TAF15 (TATA-box binding protein associated factor 15)) proteins and poly (ADP) ribose glycohydrolase (PARG). FUS stabilizes condensates, thereby preventing the spatial separation caused by the exposure of DNA double-stranded ends, and PARG regulates PAR chains within the condensates to stabilize the system. Simultaneously, FET proteins recruited to the activated condensates prevent the condensate from dissolving and promote the state of the condensates from initially rigid to dynamic, providing a pivotal role in the aggregation properties of condensates. This, in turn, gives rise to the separation of PARP1 from the end of the DNA to initiate DSB repairment. Subsequently, repair factors are recruited to unrepaired DNA ends in the condensates to perform DSB repair. This model provides a molecular and physically ordered hierarchical assembly process to explain how broken DNA ends allow the entry of repair factors, while maintaining spatial connections (Fig. 1).

To confirm that PARP1-DNA co-condensation is essential for function in cells, the authors eventually performed several cellular assays. Their cellular data showed that, for DSB repair, PARP1-DNA co-condensed to promote DNA end bridging and subsequent PARylation-dependent effectors recruitment.

In summary, this study has elucidated that PARP1 and DNA co-condense at DSB sites, which keeps the broken DNA ends spatial connection, and facilitates repair proteins recruitment, revealing key mechanisms in the DNA repair process and providing new perspectives for better understanding DNA repair. Currently, PARP inhibitors are used in the clinical treatment of cancer through a synthetic lethal mechanism,^5^ which accords with the PARP1-dsDNA co-condensation mechanism. Therefore, a comprehensive understanding of how PARP1 inhibitors regulate DNA-damaged condensates can contribute to the development of improved cancer therapies.
